# From Locus of Control to Fear: How Intolerance of Uncertainty Shapes Young Arab Adults’ Perceptions of Artificial Intelligence

**DOI:** 10.1002/puh2.70352

**Published:** 2026-08-18

**Authors:** Georges Kerbage, Camille Akkari, Rabih Hallit, Diana Malaeb, Fouad Sakr, Mariam Dabbous, Nisma Merdad, Tabassum Rashid, Rizwana Amin, Kamel Jebreen, Btissame Zarrouq, Amthal Alhuwailah, Hanaa Ahmed Mohamed Shuwiekh, Souheil Hallit, Feten Fekih‐Romdhane, Sahar Obeid

**Affiliations:** ^1^ School of Medicine and Medical Sciences Holy Spirit University of Kaslik Jounieh Lebanon; ^2^ Department of Infectious Disease Bellevue Medical Center Mansourieh Lebanon; ^3^ Department of Infectious Disease Notre Dame des Secours University Hospital Byblos Lebanon; ^4^ College of Pharmacy Gulf Medical University Ajman UAE; ^5^ School of Pharmacy Lebanese International University Beirut Lebanon; ^6^ Psychology Department College of Humanities Effat University Jeddah Saudi Arabia; ^7^ Psychology Department Al Akhawayn University Ifrane Morocco; ^8^ Department of Mathematics Palestine Technical University–Kadoorie Hebron Palestine; ^9^ Faculty of Medicine and Pharmacy Sidi Mohamed Ben Abdellah University Fez Morocco; ^10^ Department of Psychology Kuwait University Kuwait City Kuwait; ^11^ Department of Psychology Fayoum University Faiyum Egypt; ^12^ Applied Science Research Center Applied Science Private University Amman Jordan; ^13^ The Tunisian Center of Early Intervention in Psychosis Department of Psychiatry “Ibn Omrane” Razi Hospital Manouba Tunisia; ^14^ Faculty of Medicine of Tunis Tunis El Manar University Tunis Tunisia; ^15^ Department of Psychology and Education School of Arts and Sciences Lebanese American University Jbeil Lebanon

**Keywords:** fear of artificial intelligence, intolerance of uncertainty, locus of control

## Abstract

**Background:**

This cross‐sectional study examines whether intolerance of uncertainty (IU) is indirectly associated with the relationship between internal and external locus of control (LoC) and fear of autonomous robots and artificial intelligence (FARAI) in a convenience sample of Arabic‐speaking adults from four Arab countries.

**Methods:**

A cross‐sectional study was conducted in July 2023, including 1849 participants with an average age of 21.37 years, of whom 74.3% were females. Data were collected using a distributed Google Form link.

**Results:**

The results indicated that prospective anxiety was indirectly associated with the relationship between internal LoC and fear of AI. A greater internal LoC was associated with increased prospective anxiety, which, in turn, was strongly correlated with greater FARAI scores. Internal LoC was not directly related to FARAI scores. Similarly, inhibitory anxiety was indirectly associated with the relationship between internal LoC and FARAI. Greater internal LoC strongly correlated with lower inhibitory anxiety, whereas greater inhibitory anxiety strongly correlated with greater FARAI scores. Again, no direct relationship was found between internal LoC and FARAI scores. Inhibitory anxiety was indirectly associated with the relationship between external LoC and FARAI; greater external LoC correlated with greater inhibitory anxiety, which was correlated with greater FARAI scores. External LoC was not directly related to FARAI scores.

**Conclusion:**

IU, through both prospective and inhibitory anxiety, was indirectly associated with the relation between LoC and FARAI. Because the design was cross‐sectional, these findings represent associations rather than causal effects, and several of the indirect associations were small in magnitude. Tackling these forms of anxiety in young adults could be one avenue worth exploring for managing AI‐related fears.

## Graphical abstract

Intolerance of uncertainty indirectly linked locus of control to fear of artificial intelligence among Arab young adults. Prospective and inhibitory anxiety emerged as key pathways, suggesting that addressing uncertainty‐related anxiety may help reduce AI‐related fears.

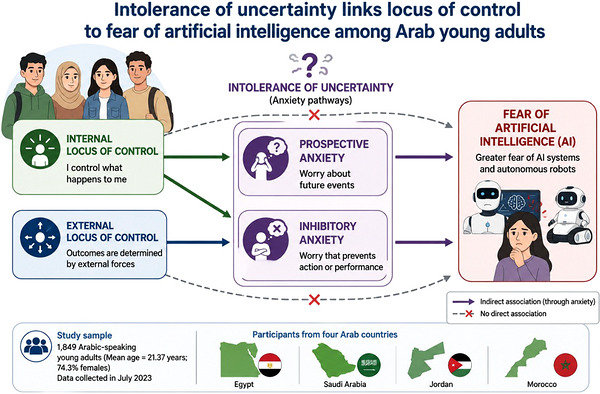

## 1 Introduction

Artificial intelligence (AI), often compared to electricity and acknowledged as a revolutionary technology [1], can be defined as the computer system's ability to autonomously complete assignments that usually demand human intelligence [2]. AI is being incorporated increasingly into people's daily lives worldwide, proving that it has transformative potential for humanity [3]. It is already a game changer in various domains, including healthcare and automobile industries. AI technologies offer several advantages; for example, AI can assist medical professionals like surgeons [4], enable users to create content like text, music, images, videos, games, and poetry [5], and help prevent accidents by identifying unsafe driving behaviors [6]. Furthermore, people increasingly rely on AI for convenience, efficiency, and better outcomes in various areas, including education [7]. Indeed, Chat Generative Pre‐Trained Transformer (ChatGPT) demonstrated a notable capability in answering various US Medical Licensing Examination (USMLE) practice questions relevant to medical knowledge and clinical reasoning [8], suggesting that AI models like ChatGPT could assist in medical training and education.

However, AI also poses significant risks, including the spread of misinformation, privacy and security issues, violations of intellectual property rights, increased bias, and potential malicious use [9, 10]. Furthermore, dependence on AI tools might lead to reduced human interaction and engagement, which are crucial in early childhood development [10]. Indeed, this overreliance can impact children's social skills and emotional intelligence, and poor‐quality AI tools could hinder learning rather than enhance it. Due to these mixed outcomes, studies have shown that individuals’ trust in and perception of AI and robots vary widely [11, 12]. Previous research suggested that people were more likely to trust AI and robots that demonstrated competence and reliability in performing tasks, suggesting that developers should focus on creating transparent and competent AI systems to enhance user trust [13]. Although AI is seen as a double‐edged sword [10, 14], some individuals recognize and are hopeful about AI's benefits; others tend to emphasize their concerns and fears regarding its drawbacks.

On the basis of research into how fear works and how it can trigger strong emotions [15], Liang and Lee came up with the term “fear of autonomous robots and artificial intelligence” (FARAI). This term describes how people expect to feel emotionally about future interactions with robots and AI, as well as how they might respond when those interactions happen [16, 17]. Importantly, fear was identified as a strong emotional reaction, making it more crucial than the simple negative attitudes toward robots and AI [16]. Factors contributing to fear included lack of familiarity with technology and concerns about job displacement and privacy, as well as negative media portrayals of robots and AI [16, 18]. Research on fear of AI has moved beyond simply labeling it as an emotional reaction and has begun to explore why some people are afraid of AI and yet still choose to use it. A recent paper addressed this question by developing a theoretical framework for AI technology acceptance, based on studies of autonomous cars, which show that fear and perceived benefits do not cancel each other out; rather, they coexist [19]. In other words, a person can be genuinely afraid of AI technology and still decide to adopt it because they weigh that fear against the benefits they expect to gain. From this perspective, FARAI is not simply a wall that blocks people from engaging in technology; it is one piece of a larger puzzle that also includes how useful people find the technology, how much they trust it, and the broader social environment they live in [19]. This framework is particularly relevant to our study because it treats FARAI as something that can shift depending on how a person perceives control and uncertainty in their life, rather than as a fixed personality trait.

In addition to the disadvantages cited above that might affect the way individuals interact with AI, research indicates that overall, individuals’ trust levels were found to be lower in AI than in humans, indicating a general skepticism toward AI systems [20]. In his 1966 study, Julian B. Rotter elaborated locus of control (LoC), a psychological concept that refers to the extent to which people believe they have influence over the consequences of events in their lives [21]. It is classified into two types. First, internal LoC refers to those who consider that their actions, choices, and efforts significantly affect the outcomes they achieve. Those people are more likely to take responsibility for their wins and mistakes and feel empowered to change their circumstances [17]. Second, external LoC relates to persons who attribute their life experiences to external causes such as chance or the influence of other people. They may believe that they have little to no influence over what they experience and that their circumstances are primarily determined by factors outside their control [21]. Therefore, internals see themselves as the architects of their own lives and think they can change their surroundings, whereas externals, on the other hand, believe they have little power to change their circumstances [20]. One's LoC orientation can vary according to many variables. For instance, parenting style can either promote independence and self‐efficacy (fostering an internal locus) or encourage conformity and dependence on external validation (leading to an external locus) [22]. Furthermore, recent studies have linked LoC to trust in various contexts. For instance, it was found that patients’ trust in their oncologists is influenced by their LoC [23]. Additionally, research indicates that externals tend to have a decreased level of trust in the medical system, which affects their adherence to treatment [24]. Furthermore, it was shown that employees with an internal LoC feel empowered to face the challenge of being replaced by robots and AI, according to the LoC theory [21]. In fact, those people are more likely to take action to minimize the potential negative effects of AI and robots, as opposed to those with an external LoC, who often feel helpless and are less inclined to address these challenges [25]. Although internals are often seen as more resilient to external influences and tend to believe that building trust among colleagues in virtual teams takes time and mutual understanding [26], past studies have found that these individuals also display higher trust and engagement in AI compared to those with an external LoC [20].

Because individuals are often unfamiliar with complex technologies, particularly AI, which can often appear unclear, this can lead to fear and a lack of trust [27]. This response may be more prominent in people with elevated levels of intolerance of uncertainty (IU), a psychological trait that is defined by difficulty dealing with circumstances that are unclear or unpredictable [28]. This trait has been studied as a binary concept linked to anxiety, notably prospective and inhibitory anxiety [29, 30]. Prospective anxiety refers to a focus on upcoming events to prepare for possible dangers [31]. It has been linked to different disorders, including generalized anxiety disorder and obsessive‐compulsive disorder [30]. In contrast, inhibitory anxiety is the tendency to hold back one's response when things are unclear. People who have elevated levels of inhibitory anxiety often feel like they cannot respond well when things are uncertain [32]. Similar to the prospective dimension, the inhibitory aspect of IU has been associated with symptoms of anxiety‐related disorders and depression [30, 33].

Individuals with high IU may feel anxious and distressed when things are not clear, which makes them want things to be clear and certain [34]. Additionally, prior research confirms that people with an external LoC exhibit elevated anxiety levels [35]. Therefore, individuals with a negative attitude toward computers and robots might not be able to handle uncertainty because they have trouble accepting the unpredictability that comes with AI technologies. Some individuals may also lack faith in AI due to concerns about their ability to replace humans in certain circumstances, such as the labor market [18]. Consequently, high anxiety levels can lead to negative attitudes toward technology and low trust in AI systems [36]. Our study will explore how IU may mediate the relationship between LoC and fear of AI.

### 1.1 The Arab Context

Although many countries globally are rapidly adopting and integrating AI into various aspects of daily life [37], several Arab nations lack clear strategies and priorities in this field. Although several governments, including Saudi Arabia, United Arab Emirates (UAE), and Qatar, have made significant advancements in planning and implementation [37], other Arab nations remain slow to adopt AI‐related technologies [17, 38]. In fact, previous studies on attitudes toward AI in the Arab world illustrated controversial results. For example, a study revealed that many Syrian healthcare personnel lack a solid understanding of AI's function and importance in healthcare, and a minority believes that AI could eventually substitute doctors [38]. Although the UAE ranked as twentieth globally and the second in the Arab world on AI implementation according to the Global AI Index 2024, a significant number of Emirati radiologists and radiographers opposed the notion that AI could influence radiology [39]. Another study revealed that many Saudi participants from various professions, including technologists, nurses, and doctors, were apprehensive about AI threatening their job security [40]. However, our understanding of the negative attitudes toward AI (such as fear) and its correlates in Arab contexts remains poor.

Interestingly, both LoC and fear can change depending on religious and cultural contexts. Cultures that emphasize individualism and self‐responsibility can promote an internal LoC. Others may emphasize collectivism and the role of community or fate, resulting in a more external LoC [41]. Within Islam, the concepts of qadar (divine predestination) and riḍā (serene acceptance of God's will) frame many life outcomes as ultimately determined by God, with human effort operating within, rather than independently of, divine will [42]. Empirical work in Muslim populations has linked stronger belief in fate to a higher external LoC, while also noting that such belief can provide a sense of inner serenity and acceptance in the face of uncertainty [43]. These culturally and religiously grounded attributional patterns may shape how young Arab adults appraise emerging and unpredictable technologies such as AI. Beyond LoC, cultural context also directly shapes how fear itself is experienced and expressed [44]. Fear and anxiety are universal emotions, yet their manifestation, intensity, and management vary considerably across cultural settings [45]. Cultural beliefs and practices shape how individuals experience and respond to fear, meaning that fear of emerging technologies like AI is not a culturally neutral phenomenon but is filtered through a population's values, worldviews, and collective experiences [44]. In collectivist cultures, for instance, fear responses tend to be more socially oriented, whereas individualistic cultures tend to frame fear as a more personal, internal experience to be managed independently [46]. For Arab populations, where collective identity, family ties, and religious worldview intersect, the fear of AI may therefore carry distinct social and existential dimensions that Western‐derived scales and frameworks may not fully capture.

### 1.2 The Present Study

On the basis of the literature above, Arab countries seem to offer a relevant context in which to study the relationship between LoC and FARAI. The present study therefore aims to examine whether IU and LoC mediate the relationship between internal LoC and FARAI in a convenience sample of Arabic‐speaking adults from four Arab countries. To our knowledge, this is the first study to examine these constructs in the Arab world.

## 2 Methods

### 2.1 Participants and Procedure

We conducted this cross‐sectional study in July 2023 and recruited a convenience sample of 1849 Arabic‐speaking young adults from four Arab countries (Lebanon, Saudi Arabia, Morocco, and Palestine). Data collection was done using a Google form link. Participants were recruited through online snowball sampling: An initial group was invited to complete the survey and to share the link within their social networks, who, in turn, shared it further. Eligibility criteria required participants to be aged 18 years or older and either citizens or residents of Lebanon, Saudi Arabia, Morocco, or Palestine. Individuals who declined participation were not included. After providing electronic informed consent, participants proceeded to complete a series of demographic questions and standardized scales. The study ensured anonymity and voluntary participation, with no financial incentives offered.

The present manuscript is based on the same participant sample as that reported in the earlier article, but it addresses different research questions and provides new analyses.

### 2.2 Sample Size Calculation

On the basis of Fritz and MacKinnon's formula, the required minimum sample size was calculated to be 413 participants [47]. The sample size estimation formula is given by *n* = *L*/*f* ^2^ + *k* + 1, where *n* is the required sample size, *L* is a tabled value corresponding to the desired power and *α* level (*L* = 7.85 for 80% power at *α* = 0.05), *f* ^2^ is the squared effect size (*f* = 0.14, a small effect), and *k* is the number of variables included in the model (*k* = 11). This yielded a required minimum of 413 participants, which our sample of 1849 comfortably exceeded.

### 2.3 Questionnaire

The questionnaire was designed to ensure anonymity and confidentiality for all participants. It was written in Arabic and contained several questions designed to collect sociodemographic data, including living area, gender, age, and country of residence. The questionnaire also included the household crowding index (HCI), defined as the number of household members (excluding newborns) divided by the total number of rooms (excluding the kitchen) [48]. Additionally, participants were also asked to complete three different scales, which aimed to evaluate psychological and social elements relevant to the study.

#### 2.3.1 Fear of Autonomous Robots and Artificial Intelligence [16]

Also known as “FARAI,” this scale is a validated tool in Arabic designed to measure individuals’ fears regarding autonomous robots and AI [17]. It is composed of four different statements, such as “Robots that can make their own decisions and take their own actions,” that participants will evaluate using a 4‐point Likert scale. The scale ranges from 1 (not afraid) to 4 (very afraid), with higher scores reflecting a greater fear of these technologies. The current reliability of the scale, measured by Cronbach's alpha, is 0.77, with a 95% confidence interval of 0.75–0.79.

#### 2.3.2 Intolerance of Uncertainty Scale (IUS‐12)

The Intolerance of Uncertainty Scale, “IUS‐12,” also validated in Arabic [49], is a shorter version of the IUS‐27 [50]. This scale includes 12 items that participants rate on a Likert scale from 1 (not at all characteristic of me) to 5 (very characteristic of me). An example item is, “I find it stressful not knowing what will happen in the future.” The IUS‐12 features two subscales that measure IU: prospective anxiety, with a current Cronbach's alpha of 0.81 (95% CI 0.80; 0.82), and inhibitory anxiety, with a Cronbach's alpha of 0.83 (95% CI 0.82; 0.84). Higher scores on this scale reflect increased levels of anxiety.

#### 2.3.3 Internal–External LoC Short Scale‐4 (IE‐4) [51]

The “IE–4,” validated in Arabic [52], employs a five‐point Likert scale ranging from 1 (applies to me to a very great extent) to 5 (does not apply to me at all). This scale consists of four items: two that measure internal LoC, such as “I'm my own boss” and “If I work hard, I will succeed,” and two that assess external LoC, including statements like “What I do is mainly determined by others” and “Fate often gets in the way of my plans.” The scores for the two subscales are derived by calculating the unweighted mean of the respective items. The current reliability for the internal LoC is 0.62 (95% CI 0.58; 0.65), whereas for the external LoC, it is 0.45 (95% CI 0.39; 0.49).

### 2.4 Statistical Analysis

Analysis was done using SPSS version 25. FARAI scores were found to be normally distributed, with a skewness of 0.176 and kurtosis of −0.475, both of which are within the acceptable range of −1 to +1 [53]. To compare means between two groups, the Student *t*‐test was utilized, whereas ANOVA was applied for comparisons involving three or more groups. Relationships between continuous variables were assessed using Pearson's correlation coefficient. Visual inspection of the regression diagnostic plots indicated that the assumptions of linear regression were met. The histogram of standardized residuals and normal P‐P plot suggested that residuals were approximately normally distributed. Moreover, the scatterplot of standardized residuals versus predicted values confirmed the assumption of homoscedasticity. Multicollinearity was also verified via variance inflation factor (VIF) values that were <5. Mediation analysis was performed using the PROCESS macro (version 3.4, Model 4) in SPSS [54], with the number of bootstrap samples set at 5000 and a 95% confidence interval. Four pathways were examined: Path A from the independent variable to the mediator, Path B from the mediator to the outcome, Path c and c′ indicating the total and direct effects from the independent variable to the outcome. In each model, internal or external LoC was entered as the independent variable, prospective or inhibitory anxiety as the mediator, and FARAI as the outcome. Adjustments were made for variables with a *p* value less than 0.25 in bivariate analyses (country, gender, living area, and the second LoC score were entered as covariates on this basis) [55]. Because all fields in the online questionnaire were required for submission, the dataset contained no missing values. Mediation significance was inferred when the bootstrap confidence interval did not include zero, with *p* values below 0.05 indicating statistical significance. Given the cross‐sectional design, these indirect effects are interpreted as indirect associations rather than as evidence of causal mediation.

## 3 Results

### 3.1 Sociodemographic and Sample Characteristics

The sample consisted of 1849 participants, with an average age of 21.37 years (SD = 3.84). Females made up 74.3% of the sample. Additional descriptive statistics are summarized in Table 1.

**TABLE 1:** Sociodemographic and sample characteristics (*N* = 1849).

Variable	*N* (%)
Country	
Lebanon	629 (34.0)
Saudi Arabia	348 (18.8)
Morocco	469 (25.4)
Palestine	403 (21.8)
Gender	
Male	475 (25.7)
Female	1374 (74.3)
Living area	
Urban	1334 (72.1)
Rural	515 (27.9)
	**Mean ± SD**
Age (years)	21.37 ± 3.84
Household crowding index (person/room)	1.39 ± 0.78
Internal locus of control	7.49 ± 1.97
External locus of control	4.85 ± 1.94
Prospective anxiety	20.65 ± 5.86
Inhibitory anxiety	12.68 ± 4.69
Fear of autonomous robots and artificial intelligence (FARAI)	5.13 ± 2.98

### 3.2 Bivariate Analysis of Factors Linked to FARAI Scores

Both Tables 2 and 3 summarize the bivariate relationships. The results indicated that residing in Lebanon, being female, and living in nonurban areas correlated with increased FARAI scores. Additionally, a greater external LoC, prospective anxiety, and inhibitory anxiety correlated with increased FARAI scores.

**TABLE 2:** Bivariate factors associated with fear of autonomous robots and artificial intelligence (FARAI) scores.

Variable	Mean ± SD	*p*	Effect size
Country		**<0.001**	0.023
Lebanon	5.63 ± 3.00		
Saudi Arabia	4.48 ± 3.02		
Morocco	5.28 ± 2.92		
Palestine	4.75 ± 2.84		
Gender		**0.006**	0.146
Male	4.81 ± 2.99		
Female	5.25 ± 2.97		
Living area		**0.030**	0.113
Urban	5.04 ± 3.02		
Rural	5.38 ± 2.85		

*Note:* Numbers in bold indicate significant *p* values.

**TABLE 3:** Pearson correlation matrix.

	1	2	3	4	5	6
1. Fear of autonomous robots and artificial intelligence (FARAI)	1					
2. Internal locus of control	0.04	1				
3. External locus of control	0.11^***^	−0.03	1			
4. Prospective anxiety	0.24^***^	0.17^***^	0.21^***^	1		
5. Inhibitory anxiety	0.20^***^	−0.04	0.34^***^	0.62^***^	1	
6. Age	0.01	−0.02	−0.01	−0.08^**^	−0.07^**^	1
7. Household crowding index	−0.02	0.07^**^	−0.06^*^	−0.004	−0.04	−0.18^***^

*p < 0.05.

**p < 0.01.

***p < 0.001.

### 3.3 Mediation Analysis

**FIGURE 1:**
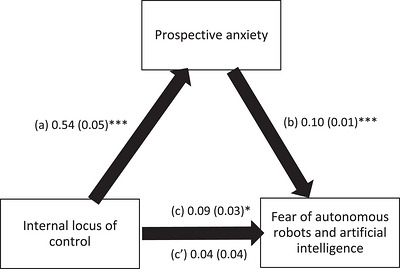
(a) Relation between internal locus of control and prospective anxiety (*R* ^2^ = 0.426); (b) relation between prospective anxiety and fear of artificial intelligence (*R* ^2^ = 0.098); (c) total effect of internal locus of control on fear of autonomous robots and artificial intelligence (*R* ^2^ = 0.077); (c′) Direct effect of internal locus of control on fear of autonomous robots and artificial intelligence. Numbers are displayed as regression coefficients (standard error). **p < 0.01; ***p < 0.001.

The mediation models were adjusted for country, gender, living area, and the second LoC score. Findings revealed that prospective anxiety was indirectly associated with the relationship between internal LoC and FARAI (Table 4). Specifically, a higher internal LoC was significantly related to increased prospective anxiety, which, in turn, correlated with more elevated FARAI scores. Notably, internal LoC did not have a direct association with FARAI score (Figure 1). This indirect association was statistically significant but small in magnitude.

**TABLE 4:** Mediation analyses results, taking internal/external locus of control as independent variables, prospective and inhibitory anxiety as mediators, and fear of artificial intelligence as the dependent variable.

Mediator	Direct effect			Indirect effect		
	Beta	SE	*p*	Beta	Boot SE	Boot CI
Model 1: Prospective anxiety as the mediator						
Internal locus of control	0.04	0.04	0.242	0.05	0.01	0.03; 0.07^a^
External locus of control	0.07	0.04	0.069	0.002	0.01	−0.01; 0.01
Model 2: Inhibitory anxiety as the mediator						
Internal locus of control	0.04	0.04	0.242	−0.01	0.01	−0.03; −0.001^a^
External locus of control	0.07	0.04	0.069	0.02	0.01	0.002; 0.04^a^

^a^ Indicates significant mediation. Direct effect refers to the direct association between internal/external locus of control and fear of artificial intelligence without the effect of the mediator, whereas the indirect effect refers to the same association through the mediators (prospective anxiety and inhibitory anxiety, respectively).

**FIGURE 2:**
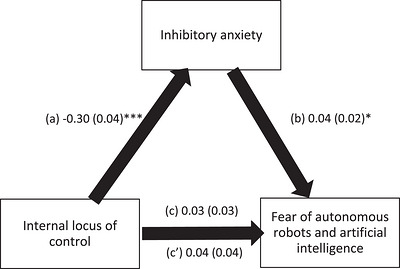
(a) Relation between internal locus of control and inhibitory anxiety (*R* ^2^ = 0.454); (b) relation between inhibitory anxiety and fear of autonomous robots and artificial intelligence (*R* ^2^ = 0.098); (c) total effect of internal locus of control on fear of autonomous robots and artificial intelligence (*R* ^2^ = 0.096); (c′) direct effect of internal locus of control on fear of autonomous robots and artificial intelligence. Numbers are displayed as regression coefficients (standard error). *p < 0.05; ***p < 0.001.

Similarly, inhibitory anxiety was indirectly associated with the relationship between internal LoC and fear of AI. Greater internal LoC was associated with lower inhibitory anxiety, which was linked to higher FARAI scores. Again, no direct association was observed between internal LoC and fear of AI (Figure 2).

**FIGURE 3:**
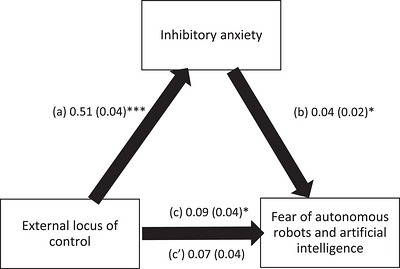
(a) Relation between external locus of control and inhibitory anxiety (*R* ^2^ = 0.454); (b) relation between inhibitory anxiety and fear of autonomous robots and artificial intelligence (*R* ^2^ = 0.098); (c) total effect of external locus of control on fear of autonomous robots and artificial intelligence (*R* ^2^ = 0.096); (c′) direct effect of external locus of control on fear of autonomous robots and artificial intelligence. Numbers are displayed as regression coefficients (standard error). *p < 0.05; ***p < 0.001.

Furthermore, inhibitory anxiety was also indirectly associated with the relationship between external LoC and FARAI (Table 4). External LoC was not directly associated with FARAI (Figure 3). As with the other models, these indirect associations were significant but modest in size and should be interpreted with the reduced reliability of the external LoC subscale in mind.

## 4 Discussion

Considered a double‐edged sword with both advantages and disadvantages, many previous studies demonstrated that people differ in their trust and attitudes toward AI and robots [56, 57]. First, our findings revealed that both internal and external LoC were indirectly associated with FARAI through the mediating variables of prospective and inhibitory anxiety. Specifically, although LoC was not directly associated with FARAI, it was associated with IU levels, which, in turn, were associated with FARAI.

Although internal LoC alone was not directly associated with FARAI and has already been linked in previous studies to a health‐promoting lifestyle and enhanced mental health [58], internals in our study reported increased levels of prospective anxiety. This form of anxiety involves a person's tendency to reduce uncertainty by actively looking for information and trying to forecast what will happen in the future [59]. The relationship between a strong internal LoC and increased prospective anxiety suggests that internals; individuals who think they can influence their outcomes [21], might experience greater anxiety about future uncertainties, including those related to AI advancements, which may be associated with greater FARAI. These individuals, as previously mentioned, may engage in constant information‐seeking behaviors, with a goal to gather as much data as possible about AI developments and their implications [59]. They may think that by gathering more knowledge, they will be better equipped to make informed decisions and reduce risks [60]. Yet, as these people learn more about the intricacies and possible repercussions of AI technology, they may encounter overwhelming amounts of information, which may be associated with cognitive overload [61]. Furthermore, as internals become more aware of the challenges related to technological advancements, this pursuit of knowledge can become a source of stress rather than relief or a positive coping strategy [62].

In contrast, internals appeared to experience lower levels of inhibitory anxiety, which relates to hesitation and, therefore, prevents them from taking action or making decisions [63]. This allows them to deal more effectively with uncertain situations, such as those involving AI [32]. Therefore, this diminished anxiety can be particularly beneficial in uncertain situations, such as those involving the implementation of AI. Indeed, previous studies showed that internals tend to have greater ambitions, are more determined, and handle challenges better [64]. As previously mentioned, these individuals are forward‐thinking and believe they can influence outcomes by their actions when confronted with uncertainty or new technologies [21]. Their proactive engagement enables them to gain knowledge and skills in addition to helping them in adapting to new technologies. By viewing challenges as opportunities for growth rather than threats, these individuals can handle uncertainty with resilience [65], making them more skilled at employing AI and other innovations in their fields. This adaptive mindset is essential for both personal and professional development. It not only supports individual development but also has a positive influence on organizations by shaping and transforming their environments [66].

These findings should, however, be considered alongside a notable methodological constraint. The internal consistency of both LoC subscales was limited, with Cronbach's alpha reaching only 0.45 for the external subscale and 0.62 for the internal subscale. Although these values are partly a consequence of the subscales containing very few items, they nonetheless introduce measurement error that may have weakened or distorted the observed associations. In particular, the findings involving external LoC, which showed the lowest reliability, should be interpreted with caution, as the subscale may not have captured the construct with sufficient precision. This may partly explain why the indirect effects involving external LoC, while statistically significant, were small in magnitude. Future studies should consider using longer, psychometrically stronger LoC instruments to obtain more stable estimates.

For instance, and bearing in mind the limited reliability of the external LoC subscale noted above, individuals who reported a strong external LoC tended to report higher levels of inhibitory anxiety. This anxiety was associated with greater hesitancy in embracing new technologies, reflecting feelings of fear and uncertainty. These individuals tend to believe that their lives are largely governed by external forces, such as powerful others or fate [21]. This tendency to avoid engaging with technologies like AI may contribute to the missing of valuable opportunities to learn, familiarize with them, and ultimately alleviation of FARAI [35, 60]. Instead, their lack of engagement may deepen their worries [67], which may make AI seem even more threatening. Thus, the findings are consistent with a heightened external LoC being associated with increased anxiety and avoidance behaviors, which, in turn, is associated with greater fears regarding AI. These pathways can waste time, since these technologies perform tasks that are time‐consuming and complex for humans to do, as well as limit personal and professional development, preventing individuals from taking advantage of the benefits that AI technology can offer [17, 27]. Additionally, their reluctance to engage with AI may slow down the overall acceptance and use of these technologies in their communities, which may, in turn, be associated with slower progress and innovation [17]. However, our findings should be interpreted with caution, bearing in mind the limited reliability of external LoC subscale noted above.

### 4.1 Limitations

First, the use of a cross‐sectional design restricts the ability to make causal conclusions. Although it is possible to identify associations between LoC, IU, and FARAI, the directionality of these relationships remains unclear. Second, given the rapid pace of AI development, perceptions and fears may evolve quickly. Third, the data collected at a single point in time may not reflect future changes in attitudes toward AI. Fourth, the study sample was mainly composed of females (74.3%) and included only young adults. This predominantly young female composition, together with the online recruitment method, may restrict the generalizability of the results to other groups, such as men or older individuals, and means the sample should be regarded as a convenience sample rather than as representative of the wider population. Fifth, our study included participants from different Arab countries, which may have cultural influences on attitudes toward technology. These cultural factors might not apply to other regions, limiting as well the applicability of the results. Sixth, relying on self‐reported tools might have caused response biases. Participants might underreport or overreport their feelings due to social desirability or lack of self‐awareness. Finally, the internal consistency of the LoC subscales was limited, which may have attenuated the observed associations and warrants caution when interpreting the findings.

### 4.2 Clinical Implications

Despite these limitations, our research provided valuable clinical implications. Given the observed indirect associations involving both prospective and inhibitory anxiety in relation to LoC and FARAI, targeted anxiety‐management approaches may be worth exploring. These programs could focus on stress‐reduction techniques and provide psychoeducation about AI to help individuals better understand and engage with this technology. Because the present study was cross‐sectional and did not test any educational or community‐level intervention, broader applications, such as curricular reform or initiatives intended to shift LoC at scale, cannot be drawn directly from these data and would need to be evaluated in dedicated, appropriately designed studies.

## 5 Conclusion

IU, through both prospective and inhibitory anxiety, was indirectly associated with the relationship between LoC and FARAI. According to the results of this cross‐sectional study, internals may experience increased prospective anxiety and decreased inhibitory anxiety, and higher scores on both dimensions of IU were associated with greater FARAI. On the other hand, and bearing in mind the limited reliability of the external LoC subscale, externals tend to report greater inhibitory anxiety, which was in turn associated with greater fears. Although our findings provide added value to the literature, further research with more diverse demographic samples and longitudinal designs is needed to enhance understanding of the dynamics between LoC, IU, and FARAI. Researchers could also investigate how cultural contexts, religiosity, resilience, and coping strategies shape perceptions of AI and its associated fears. By expanding the scope of research in these areas, we can work toward practices that promote positive interactions with AI technologies.

## Author Contributions

Souheil Hallit, Feten Fekih‐Romdhane, and Sahar Obeid involved in the study design (conceptualization). Georges Kerbage and Camille Akkari wrote the manuscript (writing – original draft). Diana Malaeb, Nisma Merdad, Tabassum Rashid, Rizwana Amin, Kamel Jebreen, Btissame Zarrouq, Fouad Sakr, Mariam Dabbous, Amthal Alhuwailah, and Hanaa Ahmed Mohamed Shuwiekh were responsible for the data collection (project administration). Souheil Hallit involved in data analysis and interpretation (formal analysis). Rabih Hallit revised the paper for intellectual content (supervision and validation). All authors approved its final version. We confirm that the authors listed on this manuscript represent the final authorship. All individuals who contributed to the study but do not meet authorship criteria have been acknowledged with their permission. All authors have read and approved the final manuscript and agree to be accountable for all aspects of the work, ensuring that questions related to the accuracy or integrity of any part of the work are appropriately investigated and resolved.

## Funding

The authors have nothing to report.

## Ethics Statement

The Ethics and Research Committee of the School of Pharmacy at the Lebanese International University approved this study protocol (2023RC‐022‐LIUSOP), which also covered a previous related study [68]. All methods were performed in accordance with the relevant guidelines and regulations (in accordance with the Helsinki Declarations).

## Consent

Submitting the form online was considered equivalent to obtaining a written informed consent.

## Conflicts of Interest

The authors declare no conflicts of interest.

## Data Availability

All data generated or analyzed during this study are not publicly available. The dataset supporting the conclusions is available upon request to the corresponding author.
